# Mitochondrial DNA Copy Number in Peripheral Blood and Melanoma Risk

**DOI:** 10.1371/journal.pone.0131649

**Published:** 2015-06-25

**Authors:** Jie Shen, Vancheswaran Gopalakrishnan, Jeffrey E. Lee, Shenying Fang, Hua Zhao

**Affiliations:** 1 Departments of Epidemiology, The University of Texas MD Anderson Cancer Center, Houston, TX 77030, United States of America; 2 Department of Surgical Oncology, The University of Texas MD Anderson Cancer Center, Houston, TX 77030, United States of America; National University of Singapore, SINGAPORE

## Abstract

Mitochondrial DNA (mtDNA) copy number in peripheral blood has been suggested as risk modifier in various types of cancer. However, its influence on melanoma risk is unclear. We evaluated the association between mtDNA copy number in peripheral blood and melanoma risk in 500 melanoma cases and 500 healthy controls from an ongoing melanoma study. The mtDNA copy number was measured using real-time polymerase chain reaction. Overall, mean mtDNA copy number was significantly higher in cases than in controls (1.15 vs 0.99, P<0.001). Increased mtDNA copy number was associated with a 1.45-fold increased risk of melanoma (95% confidence interval: 1.12-1.97). Significant joint effects between mtDNA copy number and variables related to pigmentation and history of sunlight exposure were observed. This study supports an association between increased mtDNA copy number and melanoma risk that is independent on the known melanoma risk factors (pigmentation and history of sunlight exposure).

## Introduction

Malignant melanoma is an aggressive, therapy-resistant malignancy of the melanocytes. The incidence of melanoma has been steadily increasing worldwide, resulting in an increasing public health problem [1–6]. In the United States, the lifetime risk of melanoma in 1960 was one in 600 persons; in 2000, it was one in 75 persons; and this year, it will be one in 50 persons [7]. More alarmingly, a growing fraction of patients are diagnosed at a young age but with advanced disease for which prognosis is dismal [8]. Solar ultraviolet (UV) radiation exposure, fair skin, dysplastic nevus syndrome, and a family history of melanoma are major risk factors for melanoma development [9–14]. In the past decade, various genome-wide association studies have been carried out to identify genetic variants that may contribute to the development of melanoma or melanoma-related phenotypes [15–29]. However, when those genetic and environmental factors are combined together in a risk prediction model, the overall predictive value is still less than 70%, suggesting the existence of unknown genetic or environmental risk factors for melanoma [30].

Mitochondria are semiautonomous organelles within cells that play an important role in cellular energy metabolism, the generation of reactive oxygen species (ROS), and apoptosis [31]. Compared with nuclear DNA, mitochondria DNA (mtDNA) lacks protective histones and has diminished DNA repair capacity and therefore is particularly susceptible to ROS and other types of genotoxic damage. Although the copy number of mtDNA per cell is maintained within a constant range according to the energy need of the cell to sustain normal physiologic functions, it varies significantly from 1,000 to 10,000 per cell [32]. mtDNA also significantly varies by cell type; however, in general, there is good correlation of the amount of mtDNA in various cell types. Therefore, mtDNA copy number in surrogate tissues (e.g., whole blood, etc.) might be a good indicator of mtDNA copy number in target tissues.

Some researchers have hypothesized that the variations in the mtDNA copy number reflect the net results of gene-environment interactions between unknown hereditary factors and the levels of oxidative stress (an imbalance between ROS production and the antioxidant capacity), caused by a variety of endogenous and exogenous factors, such as hormones, age, dietary and environmental oxidants/antioxidants, and reaction to oxidative damage, all of which are thought to be risk factors for various types of cancer [33–39]. This hypothesis has been supported by epidemiologic studies that demonstrated a statistically significant association between increasing mtDNA copy number in peripheral blood and increased risk of breast cancer [40, 41], non-Hodgkin lymphoma [42], chronic lymphocytic leukemia [43], lung cancer [44], renal cell carcinoma [45], pancreatic cancer [46], and colorectal cancer [47]. However, other studies showed an inverse relationship between increasing mtDNA copy number in peripheral blood and the risk of colorectal and kidney cancers [48, 49]. And no association was observed between mtDNA copy number and gastric cancer risk [50].

So far, only one study has investigated the role of mtDNA copy number in whole blood in melanoma [51]. In fact, the mtDNA copy number might be extremely relevant to melanoma because oxidative stress has been suggested to play a significant role in melanoma etiology [52–55]. Several signaling cascades such as the RAS/RAF/ERK1/2 pathway, the PI3K/AKT pathway, RAC1, and nuclear factor kappa B are involved in melanoma initiation and progression [52–55]. ROS are induced by these signal transduction cascades, and they play a fundamental role in melanomagenic processes. Cells derived from the melanocytic lineage are particularly sensitive to an increase in ROS, and melanocyte cells rely on efficient antioxidant measures. Thus, variations in mtDNA copy number may affect antioxidant capacity, influence the levels of ROS in melanocytes, and eventually contribute to melanocyte carcinogenesis. In current study, we performed the first study to investigate mtDNA copy number measured in whole blood DNA as a possible biomarker for risk of melanoma.

## Materials and Methods

### Study Participants

Detailed information on study participants has been described previously [30, 56]. In brief, patients were recruited from The University of Texas MD Anderson Cancer Center (Houston, TX). All patients with either newly diagnosed or surgically treated, histopathologically confirmed melanoma who were Texas residents and registered at MD Anderson between April 1994 and July 2013 were eligible for our study. Cancer-free participants in the control group were recruited from among unrelated clinic visitors (82.7%) and spouses (17.3%). Cases and controls were individually matched by age (±5 years), sex, and ethnicity. The exclusion criteria for patients were prior chemotherapy or radiation therapy and metastasis; the exclusion criteria for all study participants were prior cancer diagnosis (except for non-melanoma skin cancer in the patients) and any blood transfusion in the 6 months prior to recruitment. After we obtained written informed consent, each participant was given a structured self-administered questionnaire to collect demographic data and data on risk factors, such as natural hair color, eye color, skin color, the presence of moles and dysplastic nevi, their history of sunlight exposure (including freckling in the sun as a child, tanning ability, and number of sunburns), and the number of first-degree relatives with any cancer. The study protocol was approved by the Institutional Review Board at MD Anderson.

### Determination of mtDNA copy number via real-time quantitative polymerase chain reaction

The method for determining mtDNA copy number was detailed in our previous publication [41] and was shown to have high inter-assay reliability. Briefly, two pairs of primers were used in the two steps of relative quantification for mtDNA content. One pair was used to amplify the MT-ND1 gene in mtDNA. Another one was used to amplify the single-copy nuclear gene human globulin (HGB). The ratio of mtDNA copy number to HGB copy number was determined for each sample from standard curves. This ratio was proportional to the mtDNA copy number in each cell and, for each sample, was normalized to a calibrator DNA (a DNA sample from a healthy control) to standardize between different runs. All samples were analyzed in duplicate on a 96-well plate with an Applied Biosystems StepOne Plus System (Life Technologies, Carlsbad, CA). To avoid possible position effects, the polymerase chain reactions for ND-1 and HGB were always performed on different 96-well plates with the same samples in the same well positions. A standard curve of a diluted reference DNA, one negative control, and one calibrator DNA were included in each run. For each standard curve, one reference DNA sample was successively diluted 1:2 to produce a seven-point standard curve between 0.3125 and 20 ng of DNA. The R^2^ for each standard curve was 0.99 or greater. Standard deviations for the cycle of threshold value were accepted at 0.25. Otherwise, the test was repeated.

### Statistical analysis

Statistical analyses were performed using the SPSS statistical package (version 21, SPSS Inc., Chicago, IL). Differences in the distributions of demographic variables and known risk factors for melanoma obtained from the self-administered screening questionnaire between cases and controls were evaluated with chi-square tests. Crude ORs and 95% CIs for each of the known risk factors were obtained from unconditional univariate logistic regression analysis to evaluate their associations with melanoma risk **(** Table 1). To examine differences between cases and controls for the mean mtDNA copy number associated with selected categorical characteristics and differences between selected categorical characteristics for the mean mtDNA copy number within the case or control group, chi-square tests were used (Table 2). ORs and 95% CIs were estimated with unconditional multivariate logistic regression for the main effect of mitochondrial mtDNA copy number on melanoma risk. Potential confounders were tested at the P = 0.10 level, and age was the only significant confounder. The mtDNA copy number variable was examined in several ways, including as a continuous variable, as a categorical variable divided by the median value in controls, and as a categorical variable based on quartile distributions in controls (Tables 3 and 4). Cutoff points for all constructed categorical variables were based on the distribution within the control population. The dose response was tested for the quartile distribution of mtDNA copy number by inserting the mean value of each quartile and then treating the variable as a continuous variable in the logistic regression model.

**Table 1:** Distribution of selected known risk factors between melanoma cases and healthy controls.

Variables	Controls, (n = 500)	Cases, (n = 500)	OR (95% CI)
	N (%)	N (%)	
**Age (y)**			
≤60	262 (52.4)	247 (49.3)	
>60	238 (47.6)	253 (50.7)	
**Sex**			
Male	238 (47.5)	238 (47.5)	
Female	262 (52.5)	262 (52.5)	
**Hair color**			
Black or brown	388 (77.6)	320 (64.0)	1.00
Blond or red	112 (22.4)	180 (36.0)	2.05 (1.54–2.73)
**Eye color**			
Not blue	330 (66.0)	278 (53.7)	1.00
Blue	170 (34.0)	222 (44.3)	1.55 (1.19–2.02)
**Skin color**			
Dark brown	278 (55.5)	202 (40.5)	1.00
Fair	222 (44.5)	298 (59.5)	1.85 (1.43–2.39)
**Tanning ability after prolonged sun exposure**			
Average or deep	366 (73.1)	290 (58.0)	1.00
Light or none	134 (26.9)	210 (42.0)	1.98 (1.50–2.60)
**Lifetime sunburns with blistering**			
0	213 (42.6)	158 (31.5)	1.00
≥1	287 (57.4)	342 (68.5)	1.61 (1.23–2.10)
**Freckling in the sun as a child**			
No	305 (60.9)	256 (51.1)	1.00
Yes	195 (39.1)	244 (48.9)	1.49 (1.15–1.93)
**Family history of cancer**			
No	407 (81.4)	369 (73.7)	1.00
Yes	93 (18.6)	131 (26.3)	1.55 (1.14–2.13)

**Table 2:** Mean mtDNA copy number by selected known risk factors between melanoma cases and healthy controls.

	Cases (n = 500)	Controls (n = 500)	P value*
**Overall**	1.15	0.99	<0.001
**Age (y)**			
≤60	1.26	1.06	0.009
>60	1.05	0.91	0.015
*P* value^#^	0.005	0.007	
**Sex**			
Male	1.13	0.99	0.080
Female	1.17	0.98	0.034
*P* value^#^	0.534	0.672	
**Hair color**			
Black or brown	1.14	0.99	0.056
Blond or red	1.16	0.99	0.025
*P* value^#^	0.782	0.923	
**Eye color**			
Not blue	1.15	0.99	0.035
Blue	1.15	0.99	0.040
*P* value^#^	0.834	0.912	
**Skin color**			
Dark brown	1.13	0.98	0.027
Fair	1.16	1.00	0.036
*P* value^#^	0.547	0.641	
**Tanning ability after prolonged sun exposure**			
Average or deep	1.15	0.99	0.032
Light or none	1.15	0.99	0.072
*P* value^#^	0.924	0.956	
**Lifetime sunburns with blistering**			
0	1.14	0.98	0.033
≥1	1.15	1.01	0.041
*P* value^#^	0.825	0.762	
**Freckling in the sun as a child**			
No	1.15	0.98	0.048
Yes	1.15	1.00	0.046
*P* value^#^	0.843	0.826	
**Family history of skin cancer**			
No	1.13	0.95	0.026
Yes	1.20	1.07	0.034
*P* value^#^	0.012	0.024	

*The P value comparing mean mtDNA copy number between cases and controls.

^#^The P value comparing mean mtDNA copy number between groups defined by selected characteristics.

**Table 3:** Risk of melanoma as estimated by mtDNA copy number.

mtDNA index (relative copy number)	Number of cases (%)	Number of controls (%)	OR (95% CI)*
**Continuous variable**	500 (100)	500(100)	1.45 (1.12–1.97)
**Categorical variable**			
By mean in controls			
<0.99	142 (28.4)	249 (49.8)	1.00
≥0.99	358 (71.6)	251 (50.2)	2.35 (1.78–3.14)
By quartile in controls			
1st	71 (14.2)	126 (25.2)	1.00
2nd	122 (24.4)	125 (25.0)	1.73 (1.16–2.98)
3rd	137 (27.4)	123 (24.6)	1.98 (1.24–3.21)
4th	170 (34.0)	126 (25.2)	2.39 (1.53–3.42)

*ORs were adjusted by age.

**Table 4:** Joint effects between mtDNA copy number and pigmentation.

mtDNA CNV	Low (<0.99)	High (≥0.99)
**Hair color**		
Black or brown	1.00	1.57 (1.06–3.23)
Blond or red	1.46 (1.03–2.98)	3.42 (1.42–5.11)
**Eye color**		
Not blue	1.00	1.34 (1.01–1.96)
Blue	1.42 (1.03–2.76)	2.98 (1.36–4.03)
**Skin color**		
Dark brown	1.00	1.43 (1.05–1.89)
Fair	1.53 (1.06–2.85)	3.26 (1.51–4.87)

## Results

The case population included 500 patients diagnosed with histologically confirmed, non-metastatic cutaneous melanomas registered at The University of Texas MD Anderson Cancer Center. The control population included 500 cancer-free study participants who had no previous history of malignancies from among visitors (including non-blood relatives such as spouses) to the clinics by using the matching criteria of age (±5 years) and sex. Because more than 95% of study subjects were white Americans, we restricted both cases and controls to white Americans. Table 1 shows the distributions of age, sex, and selected melanoma related risk factors, including hair color, eye color, skin color, tanning ability after prolonged sun exposure, number of lifetime blistering sunburns, freckling in the sun as a child, and family history of skin cancer. As expected, the cases and controls did not differ by age or sex. In the univariate analyses, the known risk factors for melanoma were significantly associated with risk in this population. For example, study subjects who had blond or red hair, blue eyes, and fair skin had a 2.05-, 1.55-, and 1.85-fold increased risk of melanoma, respectively, compared with those who had black or brown hair, another eye color, and dark brown skin (hair color: crude odds ratio [OR] = 2.05, 95% confidence interval [CI] = 1.54–2.73; eye color: crude OR = 1.55, 95% CI = 1.19–2.02; and skin color: crude OR = 1.85, 95% CI = 1.43–2.39). Compared to average to deep tanning ability, light or low tanning ability was associated with a 1.98-fold increased risk of melanoma (crude OR = 1.98, 95% CI = 1.50–2.60). Study subjects who reported having at least one sunburn that caused blistering during their lifetime or who reported freckling in the sun during childhood had a 1.61- and 1.49-fold increased risk of melanoma, respectively (crude OR = 1.61, 95% CI = 1.23–2.10 and crude OR = 1.49, 95% CI = 1.15–1.93, respectively). In addition, having a family history of cancer (any cancer among first-degree relatives) was associated with a 1.55-fold increased risk of melanoma compared with no family history (crude OR = 1.55, 95% CI = 1.14–2.13).

The comparison of mtDNA copy number between melanoma cases and healthy controls is summarized in Table 2. Overall, melanoma cases had significantly higher levels of mtDNA copy number than healthy controls (mean: 1.15 vs 0.99, P<0.001). The difference in mtDNA copy number between cases and controls was not affected by age, sex, or selected melanoma related risk factors. When we compared mtDNA copy number within the case or control group by age, sex, and selected melanoma related risk factors, significant differences were observed according to age and family history of cancer. Regardless of case control status, older study subjects (>60 years old) had statistically significantly lower mtDNA copy number than younger subjects (≤60 years old) (P = 0.005 for the cases and 0.007 for the controls). Regardless of case control status, study subjects having family history of cancer had statistically significantly higher mtDNA copy number than subjects having no family history of cancer (P = 0.012 for the cases and P = 0.024 for the healthy controls).

The relationship between mtDNA copy number and risk of melanoma is summarized in Table 3. We first treated mtDNA copy number as a continuous variable. In the multivariate linear regression analysis, increased mtDNA copy number was associated with a 1.45-fold increased risk of melanoma after adjusting for age (adjusted OR = 1.45, 95% CI = 1.12–1.97). Second, we dichotomized mtDNA copy number into two groups (high or low) using median levels of mtDNA CNVs in controls (0.99). In the multivariate logistic regression analysis, high levels of mtDNA CNVs were associated with a 2.35-fold increased risk of melanoma after adjusting for age (adjusted OR = 2.35, 95% CI = 1.78–3.14). In further quartile analysis using 25%, 50%, and 75% values of mtDNA CNVs among control subjects as cutoff points, we found that study subjects in the 2^nd^, 3^rd^, and 4^th^ quartiles were at an increased risk of melanoma (adjusted ORs for the 2^nd^, 3^rd^, and 4^th^ categories = 1.73, 95% CI = 1.16–2.98; 1.98, 95% CI = 1.24–3.21; and 2.39, 95% CI = 1.53–3.42, respectively) when compared with those with the lowest quartile of mtDNA CNVs. A statistically significant dose–response trend was observed (P < 0.001).

Finally, we examined the joint effects of mtDNA copy number and known melanoma risk factors, including pigmentation (Table 4) and sun exposure factors (data not shown), on melanoma risk. mtDNA copy number was dichotomized into two groups (high or low) using median levels of mtDNA copy number in controls. For hair color, using study subjects who had black or brown hair and low mtDNA copy number as the reference group, those with either blond/red hair or a high mtDNA copy number alone had a 1.46- and 1.57-fold increased risk of melanoma, respectively (adjusted OR = 1.46, 95% CI = 1.03–2.98; adjusted OR = 1.57, 95% CI = 1.06–3.23, respectively), and those with both blond/red hair and a high mtDNA copy number had a 3.42-fold increased risk of melanoma (adjusted OR = 3.42, 95% CI = 1.42–5.11). For eye color, using study subjects who had an eye color other than blue and a low mtDNA copy number as the reference group, those with blue eyes, a high mtDNA copy number, or both had a 1.42-, 1.34-, and 2.98-fold increased risk of melanoma, respectively. For skin color, using study subjects who had dark brown skin and a low mtDNA copy number as the reference group, those who had fair skin, a high mtDNA copy number, or both had a 1.53-, 1.43-, and 3.26-fold increased risk of melanoma, respectively. Similar joint effects were also observed between tanning ability after prolonged sun exposure, the number of lifetime blistering sunburns, freckling in the sun as a child, and mtDNA copy number (data not shown). No significant interaction between the various risk factors and mtDNA copy number was observed in the analysis.

## Discussion

To our knowledge, this study is the first to demonstrate an association between increased mtDNA copy number and increased melanoma risk. In a previous study by Hyland et al. [51], they didn’t observe significant difference in mtDNA copy number between familial melanoma cases and family based controls. However, they found that having increasing mtDNA copy number was significantly associated with *CDKN2A* mutation status among cases. Our findings agree with those from several previous studies of various other cancers, including breast cancer [40, 41], non-Hodgkin lymphoma [42], chronic lymphocytic leukemia [43], lung cancer [44], renal cell carcinoma [45], pancreatic cancer [46], and colorectal cancer [47]. Perhaps more importantly, other studies have demonstrated an inverse relationship between increasing mtDNA copy number in peripheral blood and the risk of colorectal and kidney cancers [48, 49], and no association was observed for gastric cancer [50].

Mitochondria are the major source and target of the intracellular ROS that plays an important role in melanocytic carcinogenesis. UV exposure leads to enhanced melanocytic pigment production, which generally protects the skin from the deleterious DNA-damaging effects of UV-A and -B. However, ROS is a natural product of melanin production. The presence of melanin in cultured primary human melanocytes is associated with a higher degree of ROS accumulation and a simultaneous reduction in the cellular antioxidant glutathione [57]. The tumor suppressor protein p16^INK4A^ plays a major role in the cell cycle control of melanocytes, such that people with a familial p16^INK4A^ deficiency have a characteristic accumulation of nevi and an increased melanoma risk [58]. p16^INK4A^ is involved in the prevention of ROS accumulation in a manner that can be uncoupled from its cell cycle regulatory function. Although p16^INK4A^ deficiency was found to increase ROS levels in several cell types, the effect was particularly strong in melanocytes in a melanin-dependent manner [59]. Thus, increased ROS production in melanocytes may not only allow the accumulation of potentially oncogenic mutations in genomic DNAs but also cause significant damage on mtDNAs. Such DNA damage on mitochondria may initiate the compensatory response of increasing mtDNA production to protect the mitochondrial genome and maintain normal function. Therefore, our observation of an increase in mtDNA copy number in melanoma cases may serve as an index of the increased cellular levels of oxidative stress. Interestingly, in a recent study of the relationship between pigment production, ROS, and mitochondrial function, Boulton et al. reported a direct relationship between pigment production and the production of ROS by mitochondria, suggesting an intimate relationship among pigment production, ROS, and mitochondria [60]. In addition, fibroblasts that were exposed to mild oxidative stress showed an increase in mitochondrial mass through a cell cycle-independent pathway [61].

Since the correlation between peripheral blood mtDNA copy number and melanocyte tissue has not yet been evaluated, the biological relevance of increased peripheral blood mtDNA copy number to melanocytic carcinogenesis remains unclear. Although melanoma tumor-specific mtDNA mutations can be detected in circulation, they are more likely to be detected in advanced stage or metastatic melanoma cases [62]. Because 95% of our melanoma cases were diagnosed with early-stage melanoma (stage I and II), the contribution of tumor-specific mutations in determining peripheral blood mtDNA copy number was likely minimal, but such a potential association remains to be evaluated in future investigations. In our previous study of breast cancer, we observed inverse associations between mtDNA copy number and several antioxidants in peripheral blood [41], and a positive association between mtDNA copy number and markers of oxidative stress including thiobarbituric acid reactive substances and 8-hydroxyguanosine has been observed in humans [63]. A decrease in antioxidants leads to increased levels of oxidative stress; thus, our observation of an increase in peripheral blood mtDNA copy number in melanoma cases could be the result of decreased levels of homeostatic antioxidants, which could correlate with levels of antioxidants in melanocytes.

Considering the close relationship between pigment production, ROS, and mitochondria, our observation that peripheral blood mtDNA copy number is independent of pigmentation and history of sunlight exposure is somewhat surprising. However, as outlined earlier, peripheral blood mtDNA copy number may not reflect the mtDNA copy number in melanocytes, and the molecular mechanisms regulating mtDNA copy number might be different in peripheral blood compared with melanocytes. Future investigations are needed to assess the correlation between mtDNA copy number in peripheral blood and melanocyte tissue.

One important limitation of the current study is its retrospective design, which does not provide the power to infer a causal relationship between peripheral blood mtDNA copy number and melanoma risk. Another limitation is the quantification of mtDNA copy number in whole blood. Blood cell composition may be varied individually, and fluctuations in blood cell composition could be a confounding factor behind the observed differences. Oxygen level will affect ROS levels, and in turn, will be a stimulant to the increased biogenesis of mitochondria. In current study, blood oxygenation levels were not measured. In addition, we don’t have matched melanoma tumor tissues available to explore the correlation of mitochondrial DNA copy number between blood cells and tumor tissues. However, the study included a relatively large sample size, which provided sufficient statistical power to evaluate the association between mtDNA copy number and melanoma risk. In addition, we had detailed information on major melanoma risk factors, including pigmentation and sun exposure history. To our knowledge, this is the first investigation to demonstrate a positive association between peripheral blood mtDNA copy number and risk of melanoma. Future studies are warranted to evaluate the relative contributions of UV exposure, pigmentation, and oxidative stress-related factors and their respective influences on mtDNA copy number to provide novel insights into the biological mechanisms of mtDNA copy number variation on the development of melanoma. Additionally, we will explore the correlation the correlation of mitochondrial DNA copy number between blood cells and tumor tissues.
